# Supermatrix Phylogenetic Tree of Passerine Birds From the Indo‐Australian Archipelago Highlights Contrasting Histories of Regional Endemism

**DOI:** 10.1002/ece3.71471

**Published:** 2025-05-28

**Authors:** Audrey Miranda Prasetya, Frederick R. Jaya, Craig Moritz, Leo Joseph, Paul M. Oliver

**Affiliations:** ^1^ Division of Ecology and Evolution, Research School of Biology, and Centre for Biodiversity Analysis The Australian National University Acton Australian Capital Territory Australia; ^2^ Australian National Wildlife Collection CSIRO National Research Collections Australia Canberra Australian Capital Territory Australia; ^3^ School of Environment and Science Griffith University Nathan Queensland Australia; ^4^ Biodiversity and Geosciences Program Queensland Museum South Brisbane Queensland Australia

**Keywords:** Australia, diversity, endemism, Melanesia, passerine, phylogenetic tree, Sahul, Sunda

## Abstract

The Indo‐Australian Archipelago (IAA) is a biodiversity hotspot characterized by high levels of biotic endemism and turnover. Explanations for these biodiversity patterns tend to focus on the role of the complex and dynamic geological history of the region. However, it is only in the last decade that large‐scale phylogenetically informed analyses of macroevolutionary dynamics across the region have become feasible. A recent study of bird distributions and diversity across the archipelago highlighted marked turnover of species across geographically proximate areas in the IAA and the overarching role of sea barriers in shaping turnover. To build on this work and better understand the relative histories of bird diversification in the different areas of the IAA, we compile an updated and as‐complete‐as‐possible supermatrix phylogenetic tree for passerine birds from the region and use this to estimate and compare levels of endemism in different areas of the IAA. This genetic framework further emphasizes contrasting histories of diversification in different areas of the archipelago. As expected for this clade, we found that Australia is consistently inferred as a hotspot of paleoendemism, the islands of East Melanesia and possibly Maluku are characterized by neoendemism, while the world's largest and highest tropical island, New Guinea, is inferred to be a center of superendemism, that is, both paleo‐ and neoendemism. Our updated tree also highlights a significant increase in the number of recognized bird species across the IAA in the last 10 years, as well as improved completeness of genetic sampling.

## Graphical abstract

To better understand the relative histories of bird diversification in the different areas of the Indo‐Australian Archipelago (IAA) we compile an updated and as‐complete‐as‐possible supermatrix phylogeny for passerine birds from the IAA and use this to estimate the different levels of endemism in the IAA. We show how contrasting histories of diversification within the IAA result in different levels of phylogenetic endemism. Our updated tree also highlights a significant increase in the number of recognized bird species across the IAA in the last 10 years, as well as improved completeness of genetic sampling.

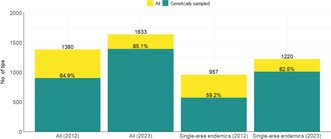

## ABSTRAK—BAHASA INDONESIA

Kepulauan Indo‐Australia (*Indo‐Australian Archipelago*/IAA) merupakan salah satu *hotspot* keanekaragaman hayati yang dicirikan oleh tingkat endemisme dan pergantian spesies yang tinggi. Penjelasan untuk pola keanekaragaman hayati ini cenderung berfokus pada peran sejarah geologi yang kompleks dan dinamis. Namun, baru dalam dekade terakhir ini analisis filogenetika berskala besar dapat dilakukan untuk analisa dinamika makroevolusi di wilayah IAA secara keseluruhan. Studi terkini tentang distribusi dan keanekaragaman burung di seluruh IAA menyorot pergantian spesies tinggi di IAA pada lokasi yang berdekatan, mengindikasikan pentingnya peran penghalang laut dalam membentuk pergantian spesies di IAA. Untuk melanjutkan studi ini dan memahami sejarah diversifikasi burung di IAA dengan lebih dalam, kami menyusun filogeni *supermatrix* yang baru dan lengkap untuk burung *passerine* dari IAA dan menggunakannya untuk memperkirakan berbagai tingkat endemisme di IAA. Kerangka genetika terbaru ini menekankan lebih jauh kontras antara sejarah diversifikasi berbagai wilayah IAA. Seperti yang diharapkan untuk burung *passerine*, kami menemukan bahwa Australia secara konsisten disimpulkan sebagai *hotspot* paleoendemisme, kepulauan Melanesia Timur dan Maluku dicirikan oleh neoendemisme, sementara pulau tropis terbesar dan tertinggi di dunia, *New Guinea* disimpulkan menjadi pusat superendemisme (paleo‐ dan neoendemisme). Pohon filogenetik kami yang baru juga menyorot peningkatan signifikan dalam jumlah spesies burung yang diakui di seluruh IAA dalam sepuluh tahun terakhir, serta peningkatan kelengkapan sampel genetika di wilayah ini.

## 1 Introduction

**FIGURE 1:**
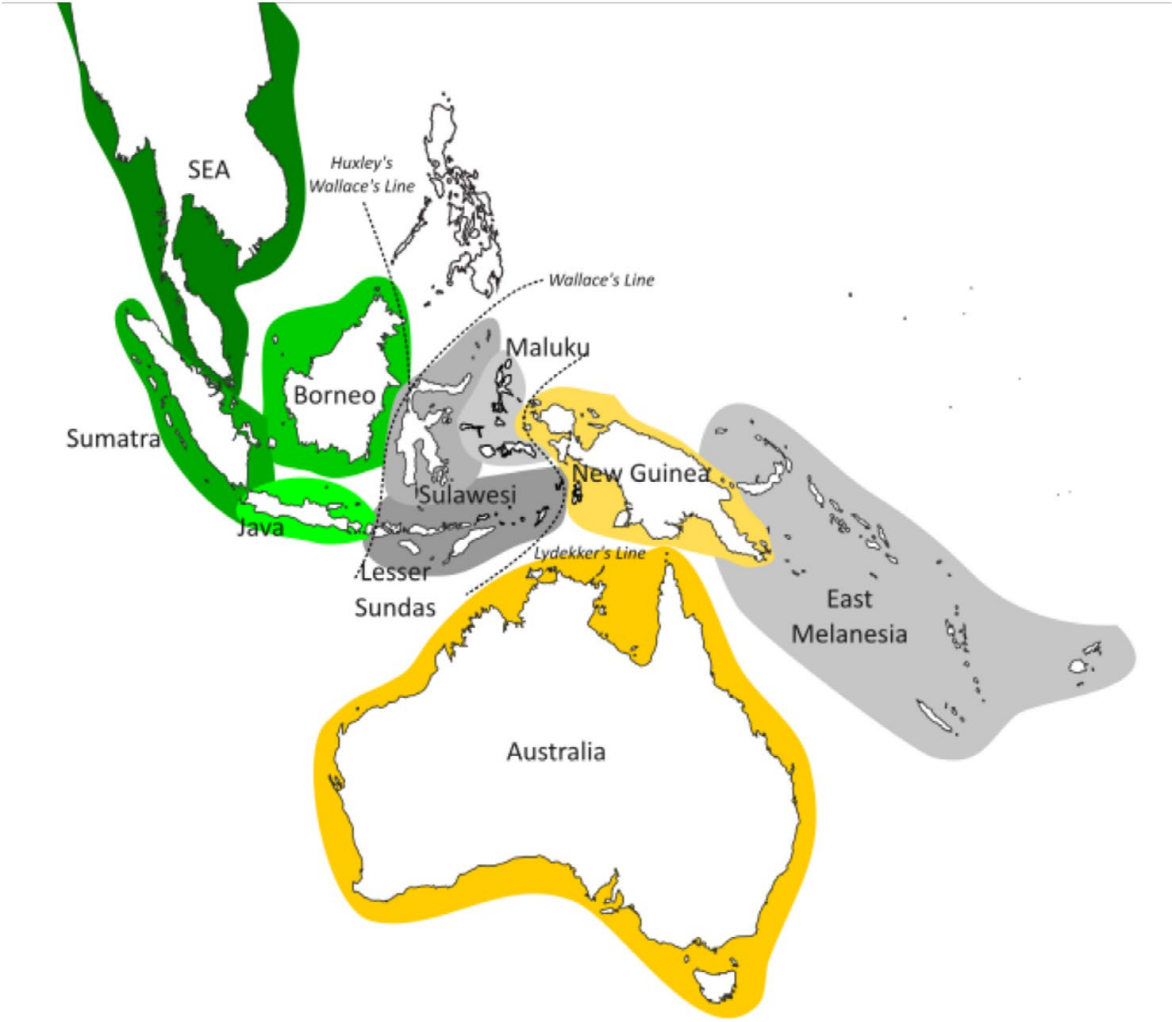
Map of the Sunda‐Sahul Biotic Interchange located in the Indo‐Australian Archipelago (IAA). The clustering of areas into three groups are represented by different color highlights: Sunda (green), Islands (gray), and Sahul (yellow). The ten areas discussed in the text are also shown.

The rich and varied biodiversity of the Indo‐Australian Archipelago (IAA) has been noted since the mid‐19th century (Huxley 1868; Wallace 1869) and it remains of immense interest to biogeographers (Jønsson et al. 2017; Joyce et al. 2020; Lohman et al. 2011; McCullough et al. 2022; Prasetya et al. 2023; Sheldon et al. 2015; White et al. 2021). The IAA (Figure 1) spans the two continental shelves of Sunda and Sahul, from the southeastern tip of continental Asia toward Australia. Marked patterns of community structuring and turnover have been noted since the 1800s—most famously, “Wallace's line” has been widely considered to mark the boundary between the lineage‐rich continental Sunda shelf and the more island‐adapted biotas that occur to the east (White et al. 2021). Given the number of islands—18,000 in Indonesia alone—and the high number of range‐restricted endemic species, the IAA offers rich potential for macroevolutionary analyses and comparative phylogeography.

Marked biotic turnover across the IAA has been characterized and widely discussed in many taxa (Lohman et al. 2011; White et al. 2021). Fewer studies have sought to compare patterns of evolutionary richness and lineage diversity across different centers of endemism spanning the IAA. Due to the mix of islands of varying age, size, history, and proximity to both other islands and putative source areas on the continent, it would, however, seem likely that differing regions might be characterized by differing histories of endemic diversification and biotic assembly. For example, many islands in the region referred to as Wallacea are thought to be relatively young (e.g., the Lesser Sundas) (Hinschberger et al. 2005). In contrast, the neighboring continental regions are more stable, older, and larger (Zahirovic et al. 2016), and hence they are predicted to hold more deep lineages. To undertake these analyses, a robust, comprehensive, and up‐to‐date phylogenetic framework is critical. Recent advances in genetic technologies have led to exponentially increasing amounts of publicly available genetic data (Kapli et al. 2020; Thomson and Shaffer 2010) that can now be accessed and downloaded to perform larger, more comprehensive, and sophisticated phylogenetic analyses (Sanderson and Driskell 2003).

Birds are one of the most well‐known and better represented taxa in phylogenetic datasets, even though the geographic region of tropical Asia and Australasia is still characterized by relatively low availability of genetic data when compared to other regions (Reddy 2014). The Indo‐Australian Archipelago (IAA) holds over ~10% of avian diversity globally. The most often used broad‐scale phylogenetic framework for birds in the IAA (and globally) is the Jetz supertree (Jetz et al. 2012) which contains “all the birds in the world” as recognized in 2012. However, only about 65% of the terminal taxa were supported by genetic data, the positions of the remaining taxa being imputed based on taxonomy. Taxonomic bias and incomplete sampling in imputed trees may result in decreased estimates of speciation or diversification rates toward the present (Marin and Hedges 2018) or introduce biases toward certain taxa or geographic regions (Rabosky 2015; Reddy 2014).

The passerines are by far the most diverse group of birds in the Indo‐Australian Archipelago and are better sampled in the region than other comparably diverse animal or plant taxa (Brady et al. 2022; Jønsson et al. 2016; Joyce et al. 2020; McCullough et al. 2022; Tänzler et al. 2016; Thomson and Shaffer 2010). Over the last decade, there has also been a marked increase in available genetic data for passerine birds from across the IAA, and the number of recognized endemic species has also increased.

Here, we synthesize these data into an updated species‐level supermatrix phylogenetic tree of passerine birds in the IAA that can be used to understand how patterns of phylogenetic depth and endemism vary across major regions. We further test the robustness of biogeographic inference from our phylogenetic tree by comparing estimates for several diversity indices derived from older supertrees (Oliveros et al. 2019). We predict that by including new phylogenetic data, we will reveal more recent radiations (neoendemism) in oceanic islands (i.e., parts of “Wallacea” and East Melanesia). Australia, as the evolutionary cradle or biogeographic origin of the oscine passerines or songbirds (Moyle et al. 2016), is predicted to be a center of paleoendemism. Finally, the island of New Guinea is of particular interest as previous studies in this region have emphasized both paleoendemism reflecting long histories of diversification either on the Australian plate (Moyle et al. 2016) or islands just to the north (Kraus and Oliver 2020; Oliver et al. 2023), and neoendemism due to the more recent orogeny (Liang et al. 2024; Roycroft et al. 2022; Toussaint et al. 2015).

## 2 Materials and Methods

### 2.1 Supermatrix Assembly

We targeted all passerine species in ten main areas spanning the IAA as per Prasetya et al. (2023), namely Australia (AUS), New Guinea (NG), East Melanesian Islands (EMN), Maluku Islands (MAU), Sulawesi (SUL), Lesser Sundas Islands (LSU), Java (JAV), Borneo (BOR), Sumatra (SUM), and Southeast Asia (SEA). For the SEA zone, we have included species from within peninsular Malaysia, Thailand, Cambodia, and Vietnam. Bird species and family‐level taxonomy follow the IOC v13.1 (Gill et al. 2020). Our original target list included passerine species from the Philippines, but we excluded taxa from this region to limit our number of areas for subsequent computationally intensive analyses and to enable us to focus studies on problematic and poorly understood regions in Wallacea and Melanesia. To understand bias in the percentage of missing taxa across areas, we calculated coverage for species that are only present in a single area (i.e., single‐area endemics, as categorized in Prasetya et al. 2023). We then assembled a species‐level matrix of Sanger sequences available from GenBank using Geneious (Kearse et al. 2012) as of April 2023. We targeted 12 loci, including four mitochondrial (cyt‐b, COI, ND2, ND3) and eight nuclear loci (c‐mos, Fib‐5, GAPDH, Myo2, ODC, RAG‐1, RAG‐2, TGFb2). For selected species of the infraorder Corvides, additional sequences with our target loci were obtained from McCullough et al. (2022). The 12 loci selected are the most widely available for birds and have been extensively used previously for avian systematics (Groth and Barrowclough 1999; Hackett et al. 2008; Jønsson et al. 2016; McCullough et al. 2022; Moyle et al. 2009; Tello et al. 2009; Thomson and Shaffer 2010). Representative sequences for each species were selected according to the following criteria: (1) sequences from a single specimen were preferred; and (2) the most recent sequencing data were preferred. If needed, individuals from multiple subspecies were included under one species to maximize the number of loci per species‐level taxon. Former subspecies that were recognized as species in IOC v13.1 but not yet updated in GenBank were treated as species. A list of all sequences used in this study for the tree is in Table S1. A mix of closely and distantly related outgroups were chosen to ensure a well‐rooted tree. Twelve outgroup taxa were chosen, including five nonpasserine birds (e.g., *Casuarius casuarius* , *Melopsittaccus undulatus*) and seven nonavian reptiles (e.g., *Chelonia mydas* , *Alligator sinensis* ). Sequences were aligned using MUSCLE (Edgar 2004) in Geneious (Kearse et al. 2012). We trimmed each locus alignment in TrimAl with a gap threshold of 0.9 (Capella‐Gutiérrez et al. 2009).

Of a total of 1633 recognized passerine species present in the IAA, we obtained sequence data for 1399 species (85.1%). There are 1220 passerine bird species from the IAA (74.4% of total) classified as single‐area endemics, and of these, 86.1% were sampled in this study. On average, each species in our dataset has a 52.9% matrix coverage (66.8% for mitochondrial loci and 45.9% for nuclear (Table S2). Less than 10% of tips are represented by only one locus and 27% of tips have high (> 10) loci) representation. Almost a quarter of sampled taxa have 100% completeness for the 12 loci. The average completeness of genetic sampling at the family level is even higher at 89.3% (87.3% mitochondrial, and 93.2% nuclear). All families are represented by at least one nuclear and one mitochondrial locus, except for rail‐babblers (Eupetidae), which lack mitochondrial loci (Table S2).

### 2.2 Phylogenetic Inference, Dating, and Grafting

To construct the IAA passerine phylogenetic tree, we adapted the backbone‐subtree grafting and dating approach from Álvarez‐Carretero et al. (2022). This approach vastly reduces the computational time and resources required for large datasets by separately dating a backbone tree and several subtrees (Álvarez‐Carretero et al. 2022). Further, this allows the monophyly of family‐level relationships as well as fossil records included in the backbone inference to be preserved despite the low loci completeness for some families. We used a recent higher level time‐calibrated phylogeny of passerines as a backbone topology and source of ~80 secondary calibrations (Oliveros et al. (2019)). This family‐level phylogeny of songbirds was derived from 4060 nuclear loci and included 137 passerine families and 13 fossils for time calibration. Then, 78 subtrees, one per family, were separately inferred using an edge‐linked proportional partition model in IQTREE2 (Minh et al. 2020). For each locus, this approach selects the best‐fitting substitution models and partitioning scheme (Chernomor et al. 2016) as determined by ModelFinder (Kalyaanamoorthy et al. 2017). Inferred topologies are shared across loci but allow branch lengths to differ. Support values for nodes in all trees were estimated using 1000 ultrafast bootstraps (Hoang et al. 2018).

Each subtree was then dated using MCMCtree (dos Reis et al. 2018; dos Reis and Yang 2011) in PAML (Yang 1997, 2007) with inferred date ranges from the Oliveros et al. (2019) backbone as calibration nodes. First, the Hessian matrix was calculated using baseml (dos Reis and Yang 2011) following steps in Álvarez‐Carretero et al. (2022) to speed up phylogenetic analyses using approximate likelihood calculations. Results of the Hessian were then used to run MCMCtree analysis separately for each family subtree using the alpha and gamma settings set in Oliveros et al. (2019). Eleven subtrees were removed after the dating analyses as they only contained one tip from the IAA (not including outgroups), yielding a final count of 67 subtrees for grafting. Every dated subtree was then grafted onto the backbone tree using an adapted version of the Álvarez‐Carretero et al. (2022) code (72sp_subtree_graft.py). To check that the final grafted tree maintains the same relative branch lengths as the ungrafted family subtrees, we compared the branch lengths from grafted and ungrafted trees per subtree (Figure S2).

As a point of comparison for downstream analysis, we also assembled two different subsets of taxa from a previously published bird supertree (Jetz et al. 2012). First, we used the part of the Jetz supertree that matches with species in our target list after taxonomic correction (hereafter termed Jetz (imputed)). This tree had ~30% of tips not based on genetic data. The second Jetz tree we included was a subset of this first Jetz tree, only including tips that were genetically sampled when this tree was estimated (hereafter termed Jetz (genetic)). An overview of the methods to obtain the three final trees that we compare in our analyses is shown in Figure S1. We compared the branch lengths of our phylogenetic tree with the other two Jetz trees as comparisons (Figure S3).

### 2.3 Phylogenetic Diversity, Phylogenetic Beta‐Diversity, and Species Richness

Species presence/absence data were assembled for ten geographic areas that can be further clustered into three groups following the convention presented by Prasetya et al. (2023): Sunda (SEA, Sumatra, Borneo, Java), Islands (Maluku Islands, Lesser Sundas Islands, East Melanesian Islands), and Sahul (New Guinea, Australia). We use the term “single‐area endemics” to describe species that are only present in one of the ten areas.

Using the presence/absence dataset and the phylogenetic trees, phylogenetic beta‐diversity (PBD) was calculated and compared with taxonomic (nonphylogenetic) beta‐diversity (TBD) using the betapart package (Baselga and Orme 2012). Phylogenetic beta diversity measures evolutionary turnover between sites (Baselga and Orme 2012). High values indicate distinct lineages across locations, often driven by historical or environmental factors, while low values suggest closely related communities. For example, regions separated by past geological events, such as the rise of mountain ranges or shifting river systems, may exhibit high phylogenetic beta diversity due to lineage divergence, whereas areas with long‐term connectivity may retain closely related assemblages and lower beta diversity.

Species richness (SR) of each of the ten areas was calculated using the pd. function from picante (Kembel et al. 2010). The package canaper (Nitta et al. 2023) was then used to calculate observed phylogenetic diversity (PD) (Faith 1992) and phylogenetic endemism (PE) (Rosauer et al. 2009). To test for statistical significance of PD and PE, observed values were compared against metrics calculated from a null model (randomized version of the community matrix; Nitta et al. 2023) resulting in two extra metrics: relative PD (RPD) and relative PE (RPE). Finally, the significance of phyloendemism for each area was estimated based on the *p*‐value calculated for RPD and RPE and using four categories: (1) palaeoendemic, (2) neoendemic, (3) mixed endemic, (4) super‐endemic (Mishler et al. 2014; Nitta et al. 2023). Areas of paleoendemism are characterized by long phylogenetic branches that have restricted ranges (significantly high RPE ratio) likely indicative of long persistence and potentially high rates of extinction. In contrast, areas of neoendemism apply to areas with range‐restricted branches (significant low RPE ratio) that are only shallowly divergent from their sister taxa, indicative of recent speciation and radiation events. Areas with concentrations of both divergent and shallow endemic lineages are characterized as areas of super endemism. All biogeographic metrics were calculated on our phylogenetic tree as well as the two Jetz trees subset mentioned above.

## 3 Results

### 3.1 Genetic and Taxon Coverage

**FIGURE 2:**
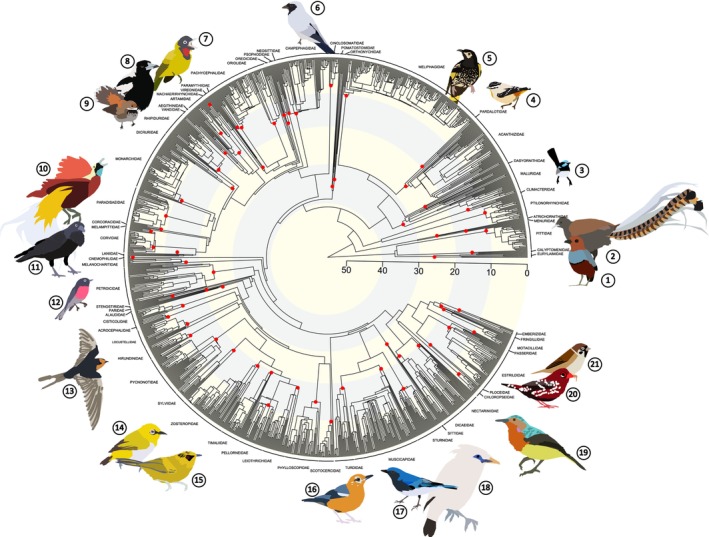
New supermatrix phylogenetic tree of all passerine birds (Order: Passeriformes) in the Indo‐Australian Archipelago. Schematic illustrations of birds by A. Prasetya. Scientific names and the corresponding bird families are listed. Secondary calibrations from the Oliveros et al. (2019) are represented by red dots.

Our final phylogenetic tree contains 1390 species tips in total (Figure 2). This is slightly less than the number of species for which sequence data were obtained due to trimming and removal of low‐quality data. The final grafted tree maintains the same relative branch lengths as the ungrafted family subtrees (Figure S2, grafted vs. ungrafted branch length scatter plot, all correlations = 1).

**FIGURE 3:**
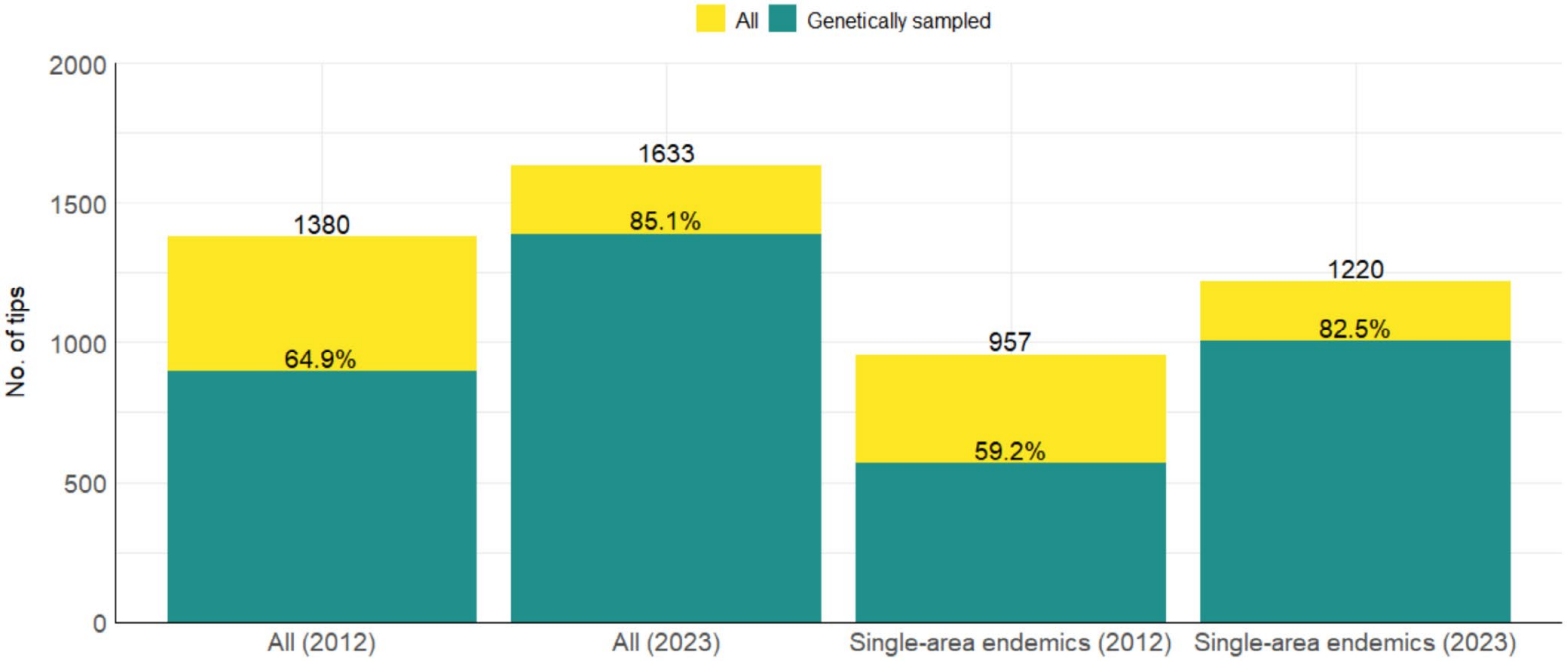
Comparisons of sampling completeness for passerine species in this study versus Jetz et al. (2012) for both the total passerine fauna and for recognized single‐area endemics. Yellow indicates the total count of species not sampled genetically and green shows the proportion genetically sampled.

When compared against Jetz et al. (2012), 564 species are different between our lists of IAA passerine species and the species sampling in the Jetz imputed tree, either due to name changes, new descriptions, or elevation of subspecies to species rank. In the Jetz et al. (2012) tree, a total of 1380 passerine bird species from the IAA are represented (tips in Jetz (imputed), and out of these, 896 were genetically sampled (Jetz (genetic)). Of the species‐level taxa recognized since 2012, most (78.3%) are island endemics (i.e., not from the Sunda Shelf or Australia). Considering differences in total recognized species, and when comparing to the Jetz (genetic) tree, genetic sampling coverage for all passerine families has greatly improved (Table S3) through an increase of over 20% in taxa for which genetic data are available (Figure 3). Particularly striking is an increase of 23.3% in the proportion of genetically sampled single‐area endemics (Figure 3).

### 3.2 Area Sampling Coverage

Sampling coverage has increased by more than 15% in all ten areas of the IAA over the last decade. The highest increases (> 30%) in available genetic data have been for passerines of New

**FIGURE 4:**
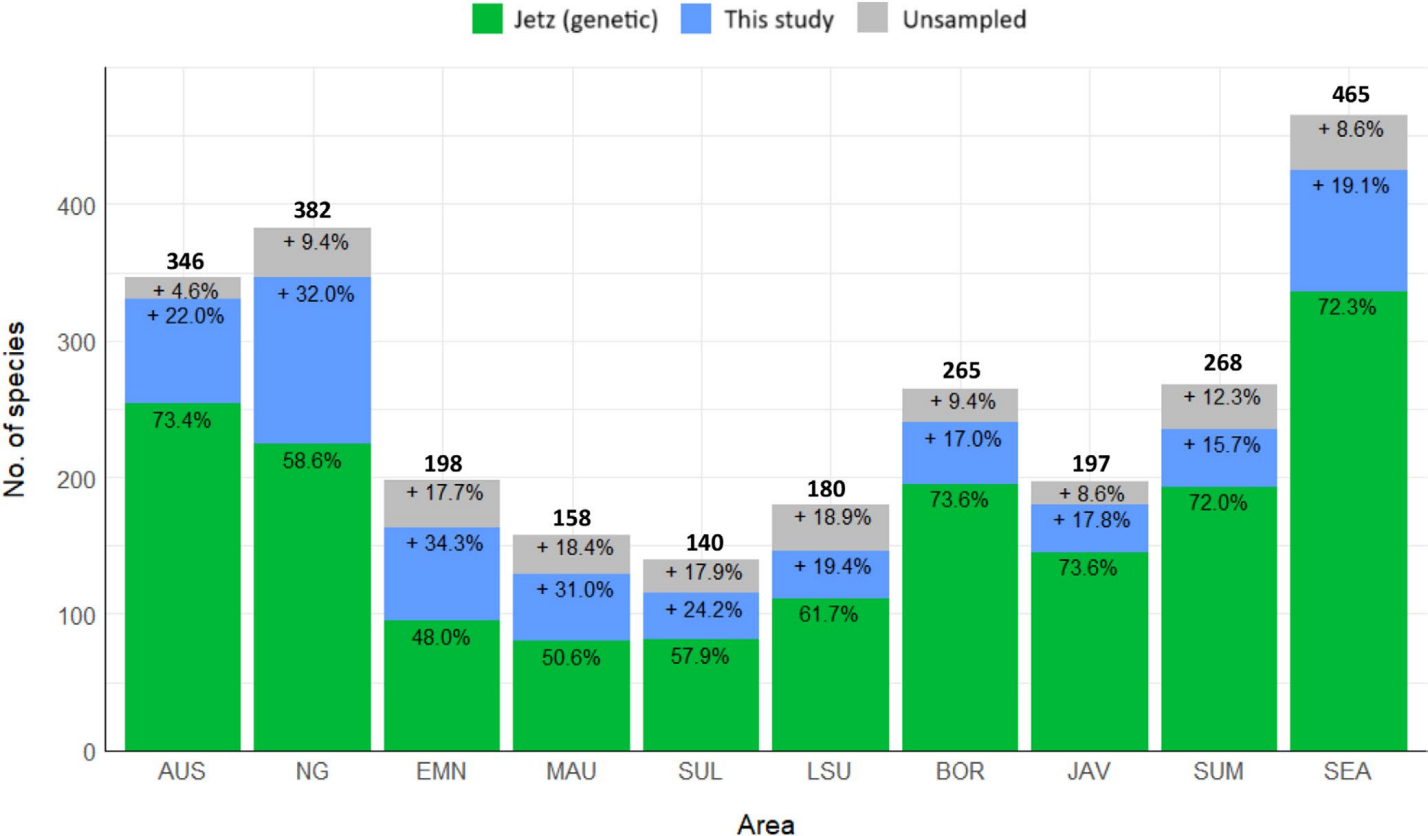
Genetic sampling coverage of recognized passerine species per area in the Indo‐Australian Archipelago as of 2023. Green shows taxa sampled in Jetz et al. (2012), blue shows taxa added in this analysis and gray show taxa for which genetic data remain unavailable. Percentages are based on taxonomy as per IOC 13.1. Total number of species per area is shown above the bar. Area codes: AUS—Australia, NG—New Guinea, EMN—East Melanesia, MAU—Maluku, SUL—Sulawesi, LSU—Lesser Sundas, BOR—Borneo, JAV—Java, SUM—Sumatra, SEA—Southeast Asia.

Guinea, East Melanesian Islands, and Maluku (Figure 4). On average, 11.7% of IAA passerine species still lack genetic data. The areas most undersampled (> 17% missing taxa) in descending order are Lesser Sundas, Maluku, Sulawesi, and East Melanesia. Australia is the best sampled region, having less than 5% missing data (Figure 4).

### 3.3 Phylogenetic Diversity and Endemism Metrics

#### 3.3.1 Beta Diversity

**FIGURE 5:**
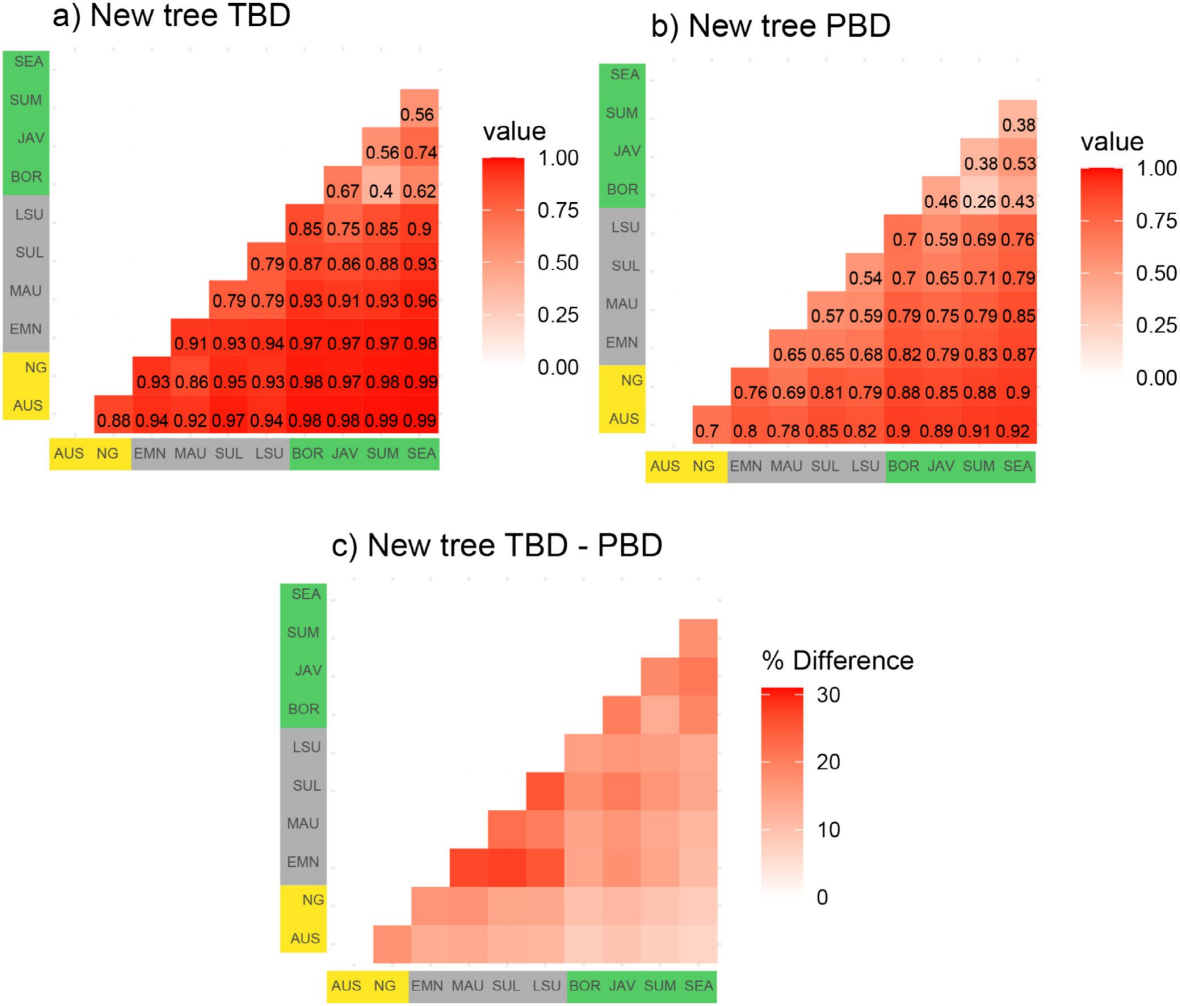
Differences in calculated pairwise taxonomic beta diversity and phylogenetic beta diversity between ten areas of the Indo‐Australian Archipelago, based on the new phylogenetic tree of passerines in the region. Area codes and region colors: Sahul in yellow (AUS—Australia, NG—New Guinea), Islands in gray (EMN—East Melanesia, MAU—Maluku, SUL—Sulawesi, LSU—Lesser Sundas), and Sunda in green (BOR—Borneo, JAV—Java, SUM—Sumatra, SEA—Southeast Asia).

Estimates of taxonomic and phylogenetic beta diversity across areas based on our phylogenetic tree are generally high (average > 0.7 overall), and the TBD tends to be slightly higher than PBD between all area pairs (Figure 5a,b). Both TBD and PBD are lowest across the areas of the Sunda Shelf (Borneo, Sumatra, Java, Southeast Asia). Area pairs that are geographically more distant produce the highest beta diversity values in all analyses (i.e., Australia vs. Southeast Asia, Sumatra vs. Australia, and New Guinea vs. Southeast Asia), emphasizing almost complete differentiation of these biotas. We also note that both TBD and PBD between Australia and New Guinea are quite high, despite belonging to the same continental shelf, a pattern which contrasts with areas that are connected within the Sunda shelf. PBD is most notably lower relative to TBD for comparisons involving areas within Wallacea, namely between varying combinations of Maluku, Sulawesi, and especially the Lesser Sundas and East Melanesia (Figure 5c).

Results of analyses using the different phylogenetic trees produced very similar results. There was a general trend for pairwise area beta‐diversity values estimated from our phylogenetic tree to be higher than estimates from the Jetz trees (Figure S4). Differences in beta diversity estimates are most marked for areas within the Sunda shelf (higher in our phylogenetic tree) and the “Wallacean” islands (lower in our phylogenetic tree). Overall, however, pairwise taxonomic and phylogenetic beta diversity calculations between areas estimated from different trees differ only slightly (< 12%) in (Figure S4).

#### 3.3.2 Phylogenetic Diversity (PD) and Phylogenetic Endemism (PE)

**FIGURE 6:**
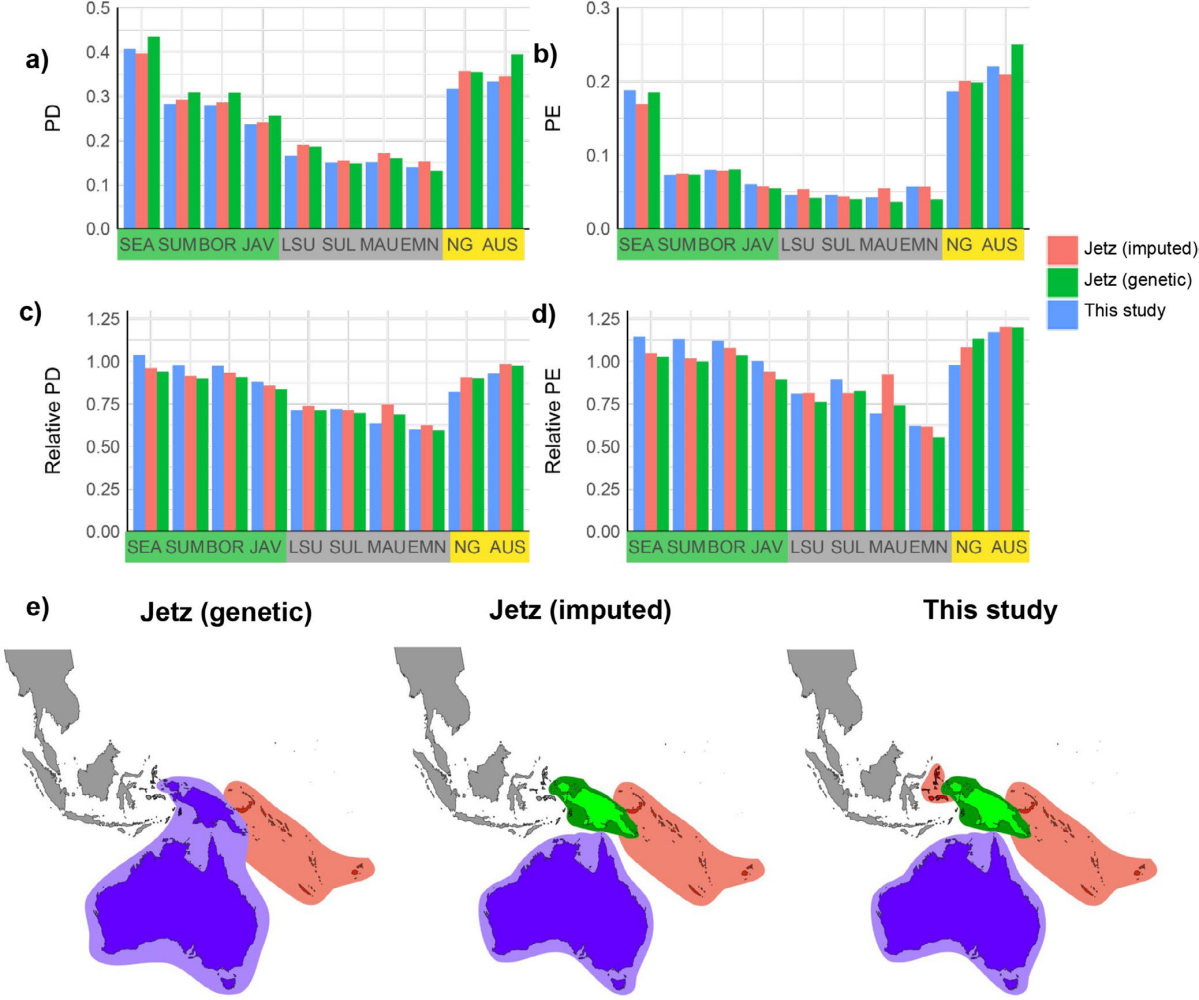
Phylogenetic diversity (PD), phylogenetic endemism (PE), relative phylogenetic diversity, and relative phylogenetic endemism for passerine birds in different areas in the IAA calculated from three different supermatrix phylogenetic datasets, namely our phylogenetic tree, the Jetz (genetic) tree; and the Jetz (imputed) tree. The maps on the right show endemism classification of area (purple = paleo‐, green = super‐, red = neo‐, gray = nonsignificant) estimated using canaper on three different phylogenetic trees. Note consistent classification of Australia and East Melanesia, and varying placement of New Guinea and Maluku.

The highest PD values are in Southeast Asia, Australia, and New Guinea. Following the three aforementioned areas are other areas within the Sunda Shelf (Borneo, Sumatra and Java), with the lowest values being in the Wallacean islands (Lesser Sundas, Sulawesi, Maluku) and East Melanesia (Figure 6a). For PE, the three areas with the highest values are the same. However, the remaining Sunda Shelf areas (Borneo, Sumatra, Java) show low PE values similar to the Wallacean islands and East Melanesia (Figure 6b). Relative PD and PE values are more similar across areas, but still noticeably lower for the small island regions of Wallacea and East Melanesia (Figure 6c,d). PD and PE estimates inferred from the Jetz tree are not markedly different from those inferred from our phylogenetic tree.

Four out of ten areas show evidence of significant endemism. Australia is categorized as paleoendemic and East Melanesia as neoendemic in all analyses based on all three phylogenetic datasets (Figure 6e and Table 1). New Guinea was classified as super endemic in both our tree and the Jetz (imputed) tree, while 69% of simulations using the Jetz (genetic) tree infer this region to be paleoendemic. Maluku was classified as neoendemic in analyses based on our tree in a small proportion of cases (21%), whereas it is nonsignificant for endemism in both Jetz trees (genetic and imputed) (Table 1).

**TABLE 1:** The results of 100 simulations on three different trees for passerine birds from the IAA, classifying endemism category using statistical comparison of PD and PE with a null model using the canaper package.

	Jetz (imputed)				Jetz (genetic only)				This study			
	Paleo	Super	Neo	NS	Paleo	Super	Neo	NS	Paleo	Super	Neo	NS
AUS	100				100				100			
NG		100			69	31				100		
EMN			100				100				100	
MAU				100				100			21	79
SUL				100				100				100
LSU				100				100				100
BOR				100				100				100
JAV				100				100				100
SUM				100				100				100
SEA				100				100				100

*Note:* Area codes: AUS—Australia, NG—New Guinea, EMN—East Melanesia, MAU—Maluku, SUL—Sulawesi, LSU—Lesser Sundas, BOR—Borneo, JAV—Java, SUM—Sumatra, SEA—Southeast Asia.

## 4 Discussion

We set out to understand phylogenetic beta diversity patterns across the passerines of the Indo‐Australian Archipelago using a species‐level supermatrix phylogenetic tree. We focused on including new genetic data and improving the representation of single‐area endemics. Our phylogenetic tree included genetic data for over 85% of passerine species from the study region—a significant increase on the ~65% coverage in the Jetz et al. (2012) supertree that we focused our comparisons against (Figure 3). We described patterns of TBD and PBD between areas in the IAA, as well as PD and PE for each area, and found evidence of super‐ and neoendemism in Melanesia, and paleoendemism in Australia.

### 4.1 New Guinea as Center of Superendemism

Perhaps the most striking result of our analyses is the classification of the vast tropical island of New Guinea as a hotspot of superendemism based on analyses of two out of our three phylogenetic datasets (Figure 6 and Table 1). This result is novel for birds but consistent with previous studies on island endemism in monocotyledonous plants, which also exhibit superendemism in New Guinea (and neoendemism in Australia, see below) (Veron et al. 2019). New Guinea superendemism unifies signatures of this region as both a museum of low diversity lineages with relatively long histories (Cámara‐Leret et al. 2020; Jønsson et al. 2011; McCullough et al. 2022; Oliver et al. 2023), and also a cradle of diversification in the last 15 Ma (Hill et al. 2023; Oliver et al. 2018; Roycroft et al. 2022; Toussaint et al. 2021). These results point to the confluence of geological and macroevolutionary processes that underpin the contemporary megadiversity of many components of the New Guinea biota (Cámara‐Leret et al. 2020—plants; Kennedy et al. 2022; Prasetya et al. 2023)—birds; Oliver et al. 2023—frogs), including initial colonization and insular diversification in the proto‐Papuan region potentially beginning as early as the Oligocene, followed by more recent rapid and extensive diversification linked to extensive island formation and orogeny since the Miocene (Roycroft et al. 2022; Tallowin et al. 2018; Toussaint et al. 2021).

### 4.2 Neoendemism Signatures for the East Melanesian and Maluku Islands

All analyses and trees also highlight neoendemism in the islands of East Melanesia, the regions to the north and east of contemporary New Guinea, and extending from the Admiralty Islands through to Vanuatu and New Caledonia. This region has never been connected by land to any continent and has been proposed as a model region for the study of bird community evolution, assembly, and extinction (Mayr and Diamond 2001; Weeks et al. 2016). Our results accord with a recent synthesis of crown ages for a suite of East Melanesian vertebrate taxa indicating the vast majority of endemic radiations are relatively young (Plio‐Pleistocene) and likely relatively recently derived from much more extensive areas of land to the west, such as New Guinea (Oliver et al. 2018). Neoendemism in these island systems may also in part reflect high turnover on relatively small and unstable island systems (Gillespie 2016; Gwee et al. 2020; Mayr and Diamond 2001). It has also been argued that relatively small islands with often unique and simplified biotic communities (including as many in East Melanesia) may provide conditions that are conducive to rapid ecological divergence and speciation (Gwee et al. 2020; McCullough et al. 2022; Moyle et al. 2016). The respective signatures of superendemism and neoendemism in New Guinea, when contrasted against East Melanesia, also further emphasize the complex confluence of geological and historical processes that underpin overall bird megadiversity across Melanesia (Prasetya et al. 2023).

The new supermatrix trees and endemism analyses provide a weak signature of neoendemism in the Maluku Islands (Figure 6e and Table 1). The islands in Maluku are also relatively small in areal extent, numerous, and geologically dynamic (Hall 2011), so this signature is not entirely surprising. However, this inference of neoendemism is not pervasive across methods or even in the one analysis where we detect it. It may be that additional sampling and taxonomic work will reinforce this signature of neoendemism in Maluku. Conversely, it may be that this region is characterized by less marked neoendemism than the more isolated and widely spread islands of East Melanesia (Mayr and Diamond 2001). We suggest that further work to understand the diversity and assembly of the biota of Maluku Islands is a potentially important area for further research.

### 4.3 Australia as Center of Paleondemism

Australia, the main landmass of the Sahul continental shelf, shows a signature of paleoendemism. This matches our expectation that diversity in this old and geologically stable continent may be relatively ancient, as well as prior knowledge that East Gondwana is the source area for most of the global diversity of oscine passerines or songbirds (Oliveros et al. 2019). When the first diversification of passerines occurred ~20 million years ago, Australia and New Guinea were quite discrete, with only much more recent (< 10 Ma) dry land connections (Harrington et al. 2017). The Sahul continental shelf, both Australia and New Guinea, harbored the oldest oscine passerines. Australia is home to a suite of early diverging low‐diversity lineages of suboscine passerines, perhaps most famously the Lyrebirds (Menuridae) and scrubbirds (Atrichornithidae) (Mitchell et al. 2021; Oliveros et al. 2019). Australia has also undergone a profound aridification over the last 20 million years (Byrne et al. 2008, 2011). This drove extensive extinctions in mesic taxa, including many that were formerly more widespread across Australia (Worthy and Nguyen 2020), and likely also drove down opportunities for accumulation of neoendemics to replace lost taxa. “Proto‐papua” is also a center of early passerine diversification but has not experienced a dramatic and widespread aridification. When compared to Melanesia (i.e., New Guinea and the East Melanesian islands) just to the north, the Australian passerine fauna may be characterized as potentially somewhat more ancient and more heavily attenuated.

### 4.4 Sunda Had No Significant Level of Passerine Endemism Detected in This Study

Given the caveat that the focus of the current study was on passerine birds, which have a deep history in Sahul, rather than Sunda, we note that our analyses inferred no signs of significant family‐level passerine endemism on specific island systems of Southeast Asia and the Sunda shelf (in contrast to the plant‐focused analyses of Veron et al. (2019). In contrast to our IAA‐restricted phylogenetic tree, a global phylogeny of birds including more taxa, especially nonpasserines, with deeper histories in the Old World such as the hornbills (Bucerotidae), broadbills (Eurylamidae) and bulbuls (Pycnonotidae) might be predicted to detect a stronger signal of paleoendemism in Sunda and Southeast Asia. Studies of other groups of plants and animals also provide abundant evidence that parts of Sunda and nearby areas of mainland Asia have a rich diversity of paleoendemic lineages (De Bruyn et al. 2014) and also served as an important source area for Australia (Oliver and Hugall 2017). Furthermore, the lack of a significant signal in endemism of the Sunda shelf in this study could also suggest the relatively large amount of shared diversity between the different areas of Sunda that we categorize separately into four areas, in contrast to the Sahul shelf that was only separated into two. On this basis, we believe that our inference of unremarkable deep time passerine endemism in Southeast Asia when compared against other parts of the IAA is likely not representative of other components of this regional biota.

### 4.5 Importance of Improving Dataset Coverage and Future Directions

Comparisons of taxonomic beta diversity and phylogenetic beta diversity across all area pairs emphasized a tendency for the latter to be lower than the former (Figure 5). This trend is particularly marked in the smaller island regions of East Melanesia, Lesser Sundas, Maluku, and Sulawesi (Figure 5). We strongly suspect that much of this trend toward lower phylogenetic beta diversity on small islands reflects the very close relationships and low divergence among shallowly divergent sister species, including many subspecies that have recently been elevated to species level or other island taxa that are only shallowly divergent (Gwee et al. 2020; Irham et al. 2020, 2023; Rheindt et al. 2020). It could be argued that this reflects a tendency to oversplit easily circumscribed small island forms. An alternative interpretation might be that small islands are prone to rapid divergence and speciation (e.g., Gwee et al. 2020). It is likely that there are elements of truth to both of these interpretations, and further investigating and contrasting these ideas could be a rich area for further research.

We also evaluated the impact of incomplete data on various diversity indices to test the robustness of some of these metrics by using three different phylogenetic datasets. Notably, both taxonomic and phylogenetic beta‐diversity produced consistent results across all three datasets. Slightly higher values of TBD and PBD in our phylogenetic tree compared to the Jetz trees for the Sunda shelf may also be attributed to the sampling of more single‐area endemic taxa (Figure 3 and Table S3). This would lead to differentiation among the areas of the Sunda shelf being more prominent. The overall robustness of diversity indices despite incomplete data is likely underpinned by several factors. First, calculations of PD are less affected by adding species that are restricted to small ranges. Furthermore, PD is blind to taxonomic rank and is impartial to shifts in diversification rate. It is worth noting that the implications of undersampled genetic data may be much more significant in other types of biogeographic and macroevolutionary analyses (Rosauer et al. 2009). For example, ancestral state reconstruction analyses could be influenced by the subdivision of species into smaller ranges (Wright et al. 2015). These considerations underscore the complex connections between taxon sampling, tree topology, and branch lengths in shaping the outcomes of diverse phylogenetic analyses. Using our tree to understand the timing and trajectory of dispersal events across the IAA is our priority for future analysis.

Our updated supermatrix phylogenetic tree for IAA passerines also highlights the vast increase in the amount of genetic and taxon coverage in the decade since Jetz et al. (2012). Mitochondrial loci remain more accessible and widely utilized than nuclear loci (Table S2). Although we have over 85% of IAA passerine taxa sampled, our average completeness across individual loci is low, especially for nuclear loci (Table S2). There may also be some species we failed to include in this phylogenetic tree not because they do not have any genetic data, but because their available genetic data are not one of our twelve targeted loci, and we are currently unaware of any such taxa. As our aim in this study was to have wide coverage of taxa rather than loci, we did not exclude taxa with only one or two loci sampled. Rather, we relied on a stricter trimming threshold to ensure a more conservative concatenated sequence was used for tree inference. However, one of the underlying challenges with such patchy data is ensuring concordance of overlapping loci sets between different species (Sanderson and Driskell 2003). Furthermore, there is still no systematic way to identify the best set of sequences for estimating a supertree. Balancing genetic and taxonomic coverage can both be issues in phylogenetic construction. The absence of sufficient loci can lead to inadequate data for robust estimation of divergence times (Marin and Hedges 2018), while reliance on only including taxa with 100% completeness is extremely difficult for large‐scale datasets that rely on data mining. Increasing the coverage of nuclear data through the use of genomic methods such as whole genomes and ultraconserved elements will strengthen the phylogenetic backbone needed to estimate relationships and timeframes of diversification (Faircloth et al. 2012). It will also aid in the resolution of species boundaries in many problematic groups of shallowly divergent species. Several significant consortia are actively working toward this aim on species‐level bird data, such as the OpenWings Consortium (https://www.openwings.org/) and the Bird 10,000 Genomes (B10K; https://b10k.genomics.cn/) Project (Feng et al. 2020; Zhang et al. 2014).

Other than increased genetic and taxon coverage, our synthesis also illustrates how work over the last decade has significantly improved geographic coverage of genetically sampled IAA passerines. Focused work on the passerine fauna of Melanesia in particular (Jønsson et al. 2011; Kennedy et al. 2022; Marki et al. 2017; McCullough et al. 2022) has led to a massive improvement in the amount of genetically available data for this region. Taxonomic changes arising from increased research on IAA birds have also led to the proliferation of single‐area endemics (Marki et al. 2017; Rheindt et al. 2020). In large part, this is indicative of the elevation of subspecies to distinct species in contemporary bird taxonomy. In this context, it is striking that the two areas we identified with the largest gaps in genetic data are Maluku and Lesser Sundas. These areas are characterized by geologically young islands (Hall 2011) and many restricted‐range species (including some that have only been described recently) (Irham et al. 2020, 2023; Rheindt et al. 2020). It is conceivable that better genetic sampling of island endemics may more strongly emphasize neoendemism in these areas. Continued efforts to both generate molecular data from historical samples and work with within‐country scientists to improve species‐level sampling will help to better elucidate the evolutionary dynamics of these regions.

## Author Contributions

**Audrey Miranda Prasetya:** conceptualization (lead), data curation (lead), formal analysis (lead), investigation (equal), methodology (equal), project administration (lead), resources (equal), validation (equal), visualization (lead), writing – original draft (lead), writing – review and editing (lead). **Frederick R. Jaya:** formal analysis (supporting), investigation (equal), methodology (equal), validation (supporting), writing – original draft (supporting), writing – review and editing (supporting). **Craig Moritz:** conceptualization (supporting), investigation (supporting), project administration (supporting), resources (equal), supervision (supporting), writing – original draft (supporting), writing – review and editing (supporting). **Leo Joseph:** conceptualization (supporting), investigation (supporting), supervision (supporting), validation (supporting), writing – original draft (supporting), writing – review and editing (supporting). **Paul M. Oliver:** conceptualization (supporting), funding acquisition (lead), investigation (supporting), project administration (supporting), supervision (lead), validation (supporting), writing – original draft (supporting), writing – review and editing (supporting).

## Conflicts of Interest

The authors declare no conflicts of interest.

## Supporting information


Data S1.

Data S2.

## Data Availability

Data and code used for this study are available at: https://figshare.com/s/ac2139525d85fa12d923.
