# Supplementary material for: Supermatrix Phylogenetic Tree of Passerine Birds From the Indo‐Australian Archipelago Highlights Contrasting Histories of Regional Endemism
Source: Ecol Evol. 2025 May 28;15(6):e71471. doi: 10.1002/ece3.71471 (PMC12119147; doi:10.1002/ece3.71471)
Supplement: Supplementary file 1 — Data S1. [file ECE3-15-e71471-s002.docx]

**SUPPLEMENTARY FIGURES**

**
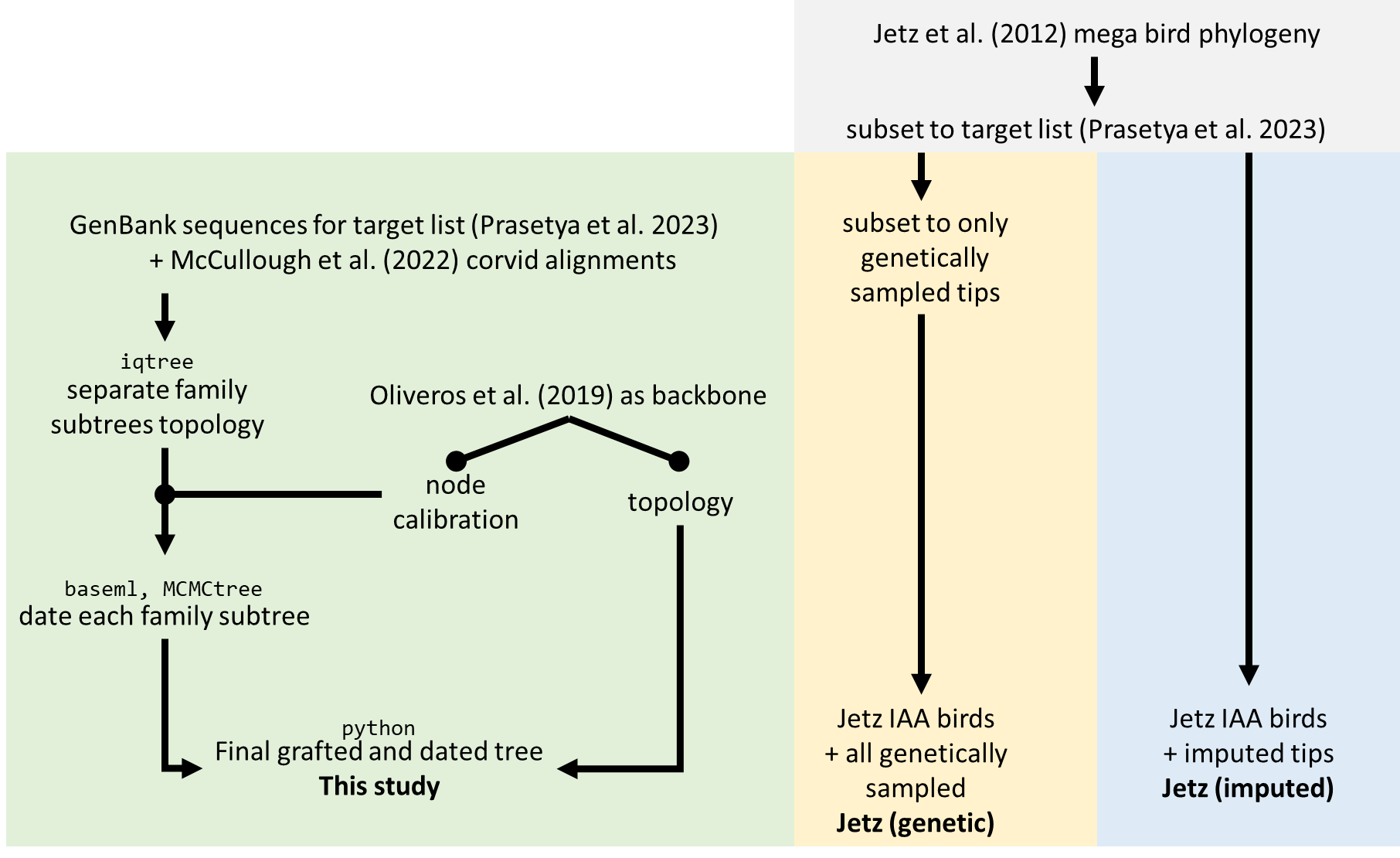
**

**Figure S1.** Overview of methods for obtaining the three trees of passerines in the Indo-Australian Archipelago used in this study. The focal tree produced in this study (‘This study’) used genetic samples mined from GenBank and McCullough et al. (2022). A phylogenetic tree was then created using a backbone-subtree approach by combining the new genetic data with a family-level passerine tree. Two phylogenetic trees subsetted from the Jetz et al. (2012) mega bird phylogeny was used to compare biogeographic inferences-based trees of varying completeness. One tree included all tips in the Jetz phylogenetic tree correcting for taxonomic changes and includes imputed tips (‘Jetz (imputed)’), while the other contained a subset of this tree where all tips are genetically sampled (‘Jetz (genetic)’).

**Figure S2.** Comparison of grafted vs ungrafted edge length for a select few of family-level subtrees. All remaining subtrees can be found in the Appendix.


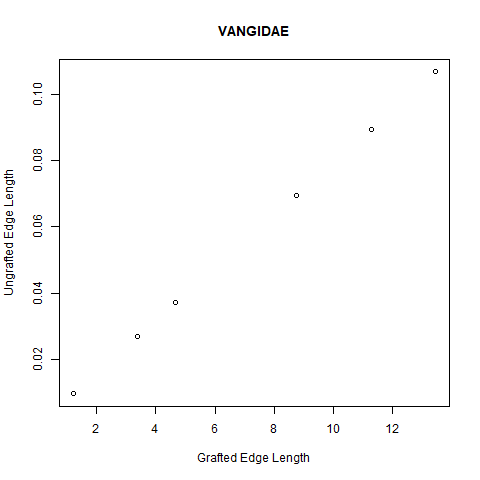

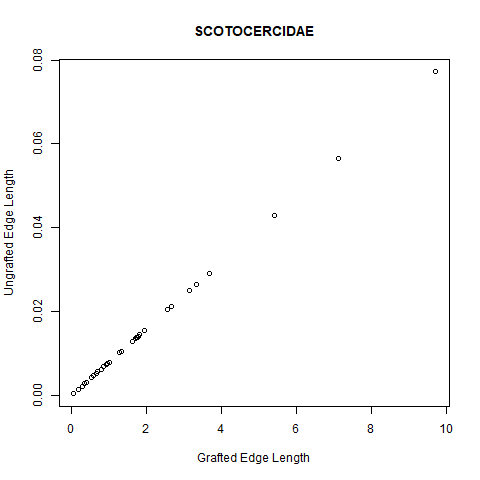

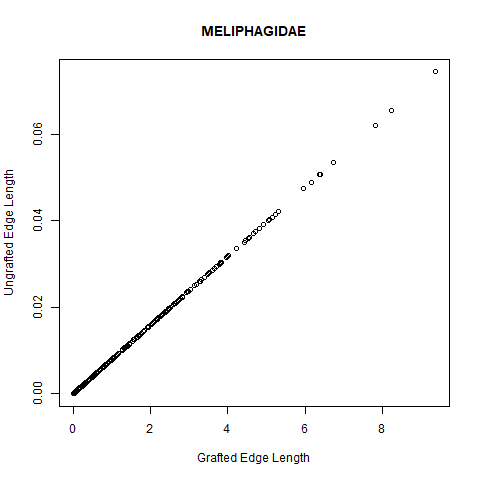

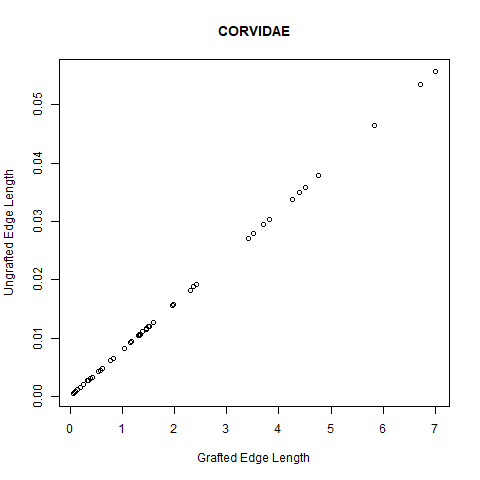

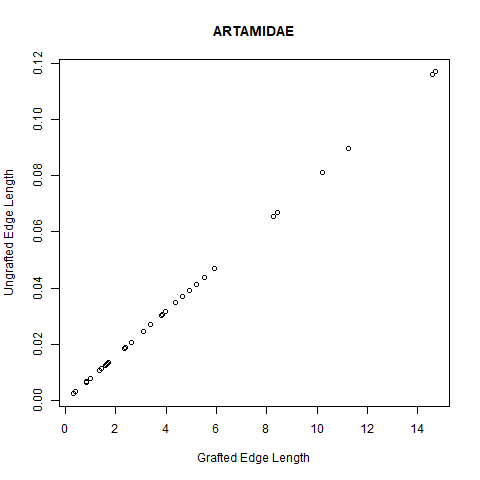

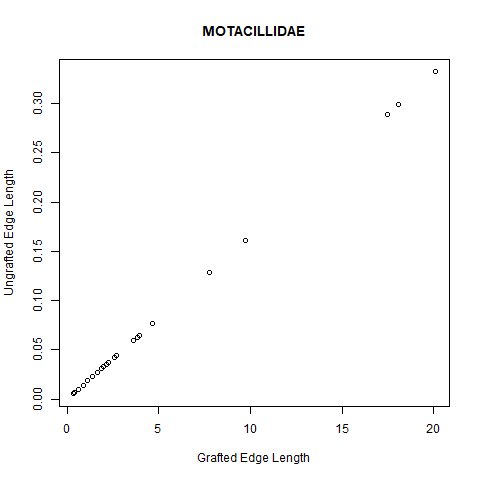


**
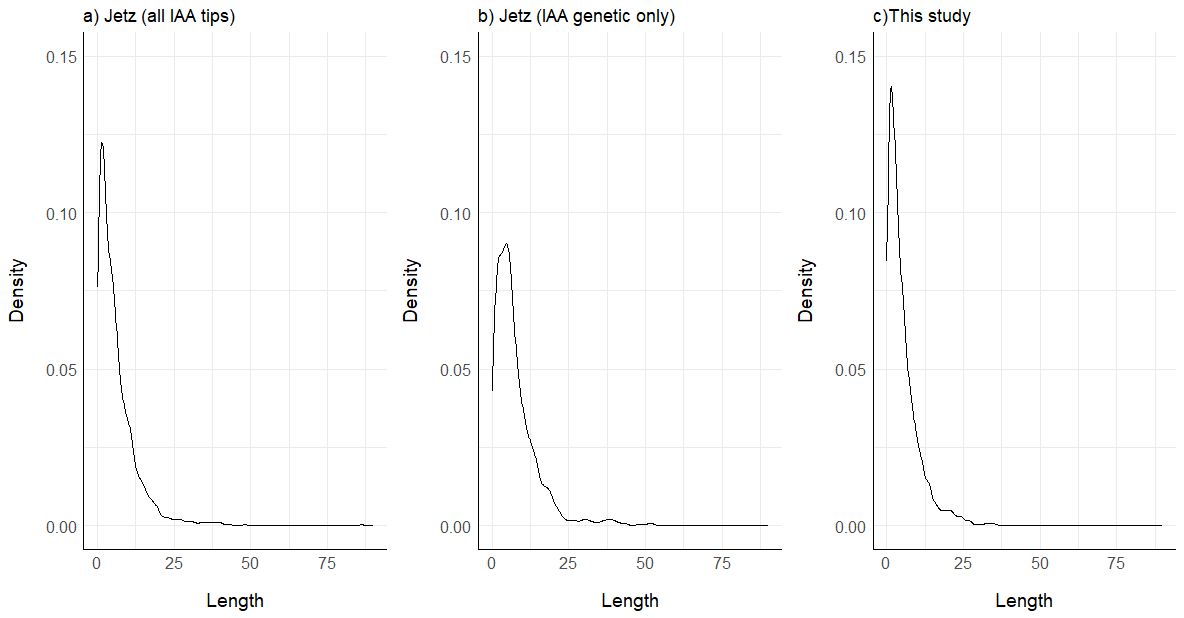
Figure S3.** Density plot of branch lengths comparing the three different phylogenies of passerine birds in the Indo-Australian Archipelago.


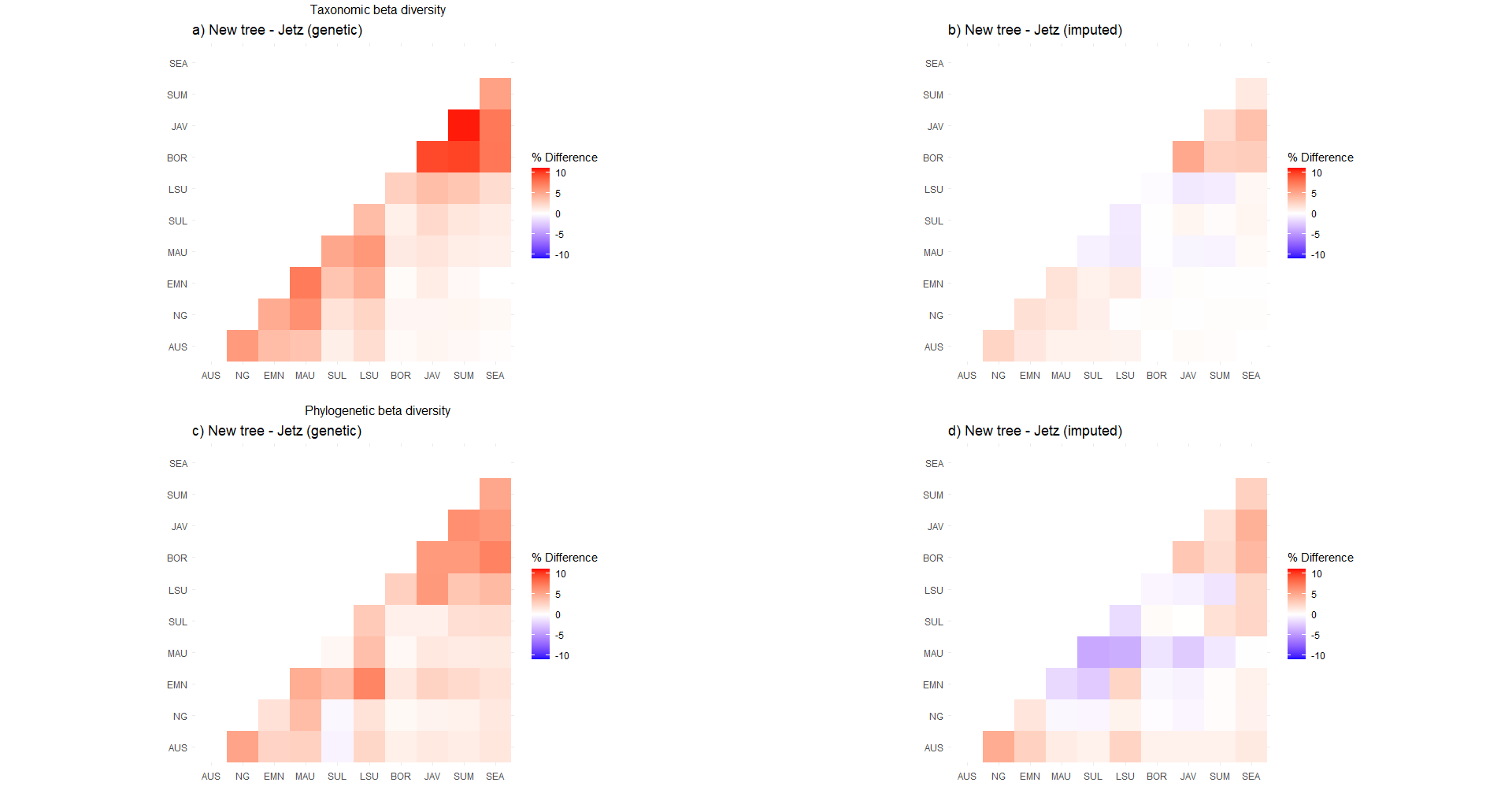

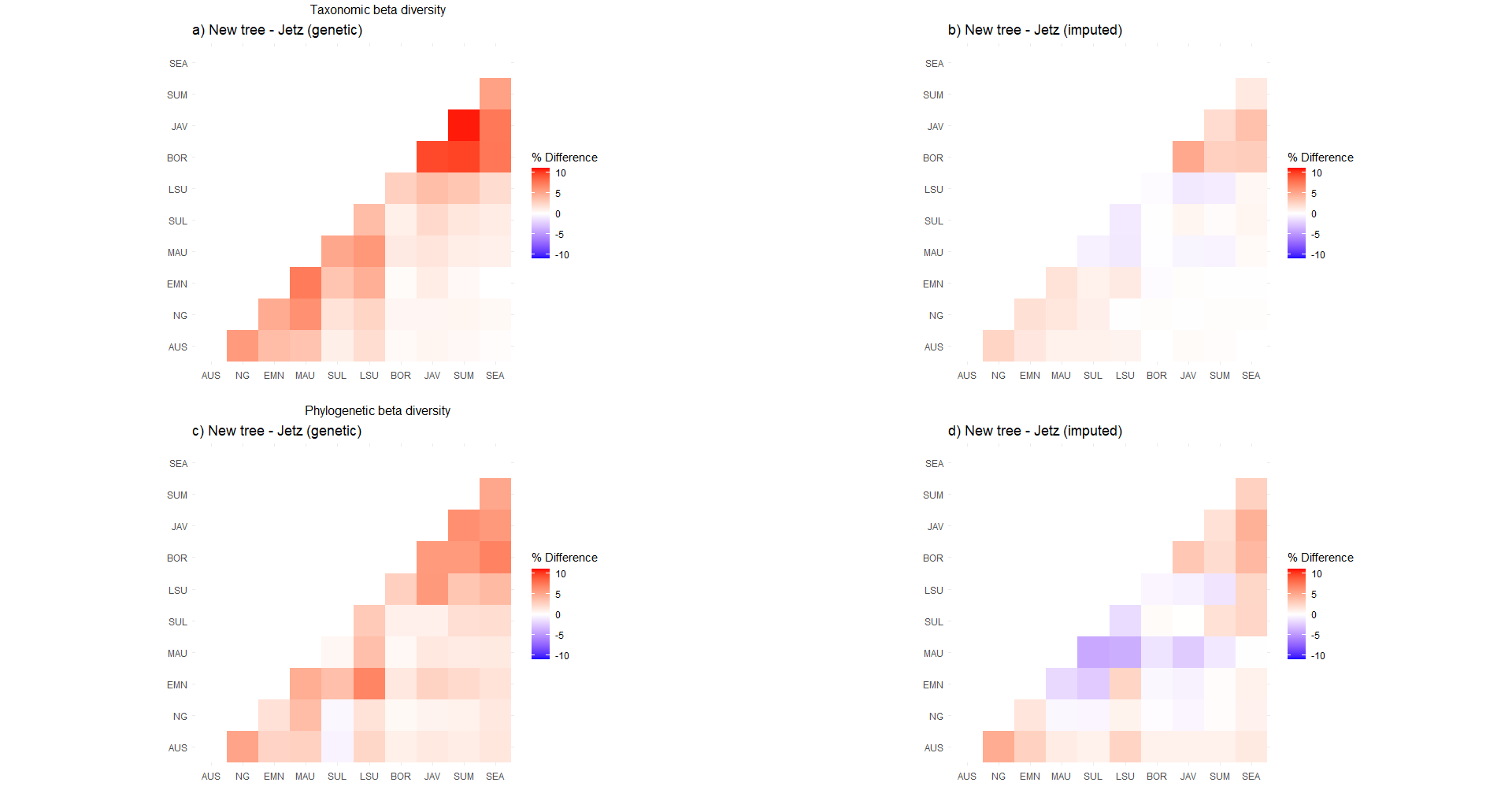


**Figure S4.** Differences in calculated pairwise taxonomic beta diversity and phylogenetic beta diversity of ten areas in the Indo-Ausralian Archipelago, between our new passerine phylogenetic tree and two different subsets of Jetz et al. (2012) phylogeny.
